# Meta-meta-analysis on the effectiveness of parent-based interventions for the treatment of child externalizing behavior problems

**DOI:** 10.1371/journal.pone.0202855

**Published:** 2018-09-26

**Authors:** Tanja Mingebach, Inge Kamp-Becker, Hanna Christiansen, Linda Weber

**Affiliations:** 1 Department of Child and Adolescent Psychiatry, Psychosomatics and Psychotherapy, Marburg, Germany; 2 Department of Clinical Child and Adolescent Psychology, Philipps University Marburg, Marburg Germany; Universite de Bretagne Occidentale, FRANCE

## Abstract

**Objective:**

The aim of this study is to perform the first meta-meta-analysis on the effectiveness of parent-based interventions for children with externalizing behavior problems. Even though parent-based interventions are considered as effective treatments the effects reported in meta-analyses are heterogeneous and the implementation in clinical practice is suboptimal. Recapitulative valid effect predictions are required to close the still existing gap between research findings and clinical practice. The meta-meta-analytic results on changes in child behavior shall result in a clear signal for clinical practice.

**Methods:**

This meta-meta-analysis encompasses 26 meta-analyses identified via search in electronic databases (PsycINFO, Medline, PubMed). Meta-analyses had to report effects of parent-based interventions on child behavior and focus on children under the age of 13 years with externalizing behavior problems in a clinical setting. Analyses were based on random-effects models. To combine results, the effect estimates of the meta-analyses were transformed to SMD and weighted to correct for primary study overlap. The meta-meta-analysis is registered on PROSPERO, registration number CRD42016036486 and was conducted in accordance with the Preferred Reporting Items for Systematic reviews and Meta-Analyses statement (PRISMA).

**Results:**

The results indicate a significant moderate overall effect for child behavior (SMD = 0.46) as well as for parent reports (SMD = 0.51) and observational data (SMD = 0.62). Further analyses focusing on child externalizing behavior yielded significant and moderate effects (SMD = 0.45). All effects remained stable to follow-up. Considerable heterogeneity was observed within results.

**Conclusion:**

Parent-based interventions are shown to be effective in improving behavior in children with externalizing behavior problems, as assessed using parent reports and observational measures. The present results should encourage health care providers to apply evidence-based parent-based interventions.

## Introduction

Externalizing behavior disorders constitute one of the major reasons for the referral of children to mental health agencies [1]. Rates of externalizing disorders in preschool children are similar or even slightly higher than in older age [2,3]. Due to the high prevalence rates and long-lasting impairment associated with an early beginning of disruptive behaviors [4,5], effective interventions are needed that target children at risk of disturbed developmental processes.

The role of parents is essential in the complex interaction concerning child development. Parents need to exhibit effective parenting behaviors as well as responsiveness and sensitivity to child signals and behaviors to facilitate socialization processes in children [6]. Moreover, parenting practices such as inconsistent discipline or negative emotional expressiveness negatively affect child emotion regulation, which in turn can lead to disruptive behavior problems [7]. In particular, cycles of escalating coercive child-parent interactions as well as harsh and inconsistent parenting practices contribute to the development and maintenance of behavior problems [8,9]. Effective parenting behaviors that enhance children’s self-regulatory skills, facilitate prosocial behaviors, and that are related to preventive effects on behavior problems are therefore essential.

Parent-based interventions specifically target these relevant parental behaviors. For the treatment of externalizing behavior problems there is evidence that parent-based interventions show positive effects on parental characteristics such as parenting behavior, parental perceptions and parental mental health [10] and improve child behavior [11–13]. However, despite the broad data base, the implementation in clinical practice and access to parent-based interventions is limited [14]. Furthermore, effects of parent-based interventions are heterogeneous, and range from small to large effect sizes making estimation of effects difficult [10]. This variation in meta-analytic effects may be caused by methodological and content-related heterogeneity [15]. Results can vary depending on the source of the rating [9] and the definition of the outcome category. Furthermore, the inclusion criteria differ between the studies, and the studies include different populations (e.g. clinical vs. non-clinical trials), interventions, or study designs.

Additionally, effects of parent-based interventions on child behavior are mostly assessed by parent report, which raises doubts about the reliability of these results [16]. Positive attributions of parent-based interventions may emerge, as otherwise, the invested costs are perceived as being too great [17]. That is why more recent studies take into account observational measures as a more objective source of information on child behavior. Those indicate a stable small to moderate effect of parent-based interventions on child behavior, inflicting further heterogeneity.

### Aims of this study

To clarify the magnitude of effects and to facilitate the implementation of parent-based interventions in clinical practice, the aim of the present study is to conduct a comprehensive review and meta-meta-analysis summarizing all existing meta-analytic estimates concerning the impact of parent-based interventions on child behavior in a population of children with externalizing behavior problems. The effects on parental characteristics will be presented elsewhere [18].

The term externalizing behavior problems includes children with externalizing behavior problems and diagnosed disorders as conduct disorder (CD), oppositional defiant disorder (ODD), and attention-deficit hyperactivity disorder (ADHD). This broad understanding reflects a dimensional conceptualization of externalizing behavior problems as well as the relevant diagnostic classifications and there is evidence for this conceptualization [19–21]. Surely this dimensional understanding of externalizing behavior problems raises the risk of heterogenous populations and the inclusion of results from a preventive setting while this review focuses on effects in a clinical setting. Nevertheless, the opportunity of including a larger database and the closeness to clinical practice are a strong argument in favor of including both children diagnosed with disorders as ODD, CD and ADHD as well as children described as presenting with externalizing behavior problems.

Meta-meta-analyses (also called second-order meta-analyses) are meta-analyses of meta-analyses. They apply similar techniques to meta-analyses of primary studies in order to combine results of meta-analyses [22–25]. In a first step, the effects of parent-based interventions on child behavior are summarized irrespective of the source of information. In a second step, effects on child behavior are analyzed separately for parent report and observational data. Furthermore, a third analysis is restricted to meta-analyses explicitly reporting results on externalizing behavior problems alone. All displayed analyses are conducted for post and follow-up measures. To reach an unbiased evaluation of the effectiveness of parent-based interventions, overlap of primary studies in different meta-analyses is taken into account.

## Methods

The meta-meta-analysis is registered on PROSPERO, registration number CRD42016036486 (S2 File) and was conducted in accordance with the Preferred Reporting Items for Systematic reviews and Meta-Analyses statement (PRISMA, S1 Table).

### Inclusion criteria

Meta-analyses were eligible if they measured the efficacy of parent-based interventions for preschool- and school-aged children with externalizing behavior problems (see above). They had to focus on parent-based interventions for children with mental health disorders (ODD, CD, ADHD) or externalizing behavior problems and could not be conducted solely in a preventive setting. At least one child behavior outcome had to be reported. Meta-analyses published in English and German were considered.

The following two outcome categories were stated: (1) child behavior overall defined as a broad child behavior outcome including positive behaviors (e.g. prosocial), externalizing behavior problems (e.g. noncompliance, aggressive behavior, disruptive behavior) and internalizing symptoms (e.g. anxiety, depressive symptoms); consequently measures of any kind of non-performance-related child behavior were considered, on the one hand global measures as e.g. Child Behavior Checklist (CBCL), Dyadic Parent-Child Interaction Coding System (DPICS), Teacher Assessment of Social Behavior (TASB) and on the other hand specific measures as e.g. Children’s Depression Inventory (CDI), Social Behaviour Questionnaire (SBQ) (2) externalizing child behavior defined as solely disruptive behaviors that violate social norms (e.g. noncompliance, aggressive behavior); only measures restricted to externalizing behavior problems were considered, e.g. Eyberg Child Behavior Inventory (ECBI), Child Behavior Checklist-Externalizing (CBCL-E), Strengths and Difficulties Questionnaire Conduct Scale (SDQ-Conduct).

### Search strategy

A systematic search in different electronic databases (PsycINFO (EBSCO), Medline (OVID), PubMed) was undertaken in March 2016 and updated in April 2018 to determine relevant meta-analyses. The following search terms were employed to look for relevant meta-analysis: *meta-analysis* AND parent* training OR parent* intervention OR parent* program AND *children* OR *preschool* OR *toddler* OR *childhood* OR *infant*. In the databases PubMed and Medline all fields were searched. In the database PsycINFO only the abstracts were searched, because searching all field was not selective enough. No further limits were set. The full electronic search strategy is illustrated in Appendix A in S1 File.

### Screening of records and data extraction

Eligibility assessment was independently realized by two reviewers (LW, TM). Disagreements were subsequently resolved by consensus. First, all abstracts were screened (n = 297) and the following aspects led to exclusion of studies (n = 220): no meta-analysis; no reference to topic of interest; children with disorders/problems other than disruptive behavior problems (e.g. autism spectrum disorders, physical impairment); merely preventive interventions; publication in a language other than English or German; full text not available; update of article available. Furthermore, unpublished studies were excluded.

In a next step, screening of the full texts of the remaining articles was undertaken (n = 77) and the following aspects led to exclusion: a main focus other than the topic of interest (n = 22); sample too specific (e.g. teenage parents) or topic too specific (e.g. home visitation) (n = 7); not enough statistical information (n = 9); no outcomes of child behavior (n = 3); merely preventive interventions (n = 8). We placed our focus on face-to-face parent training interventions to obtain maximum homogeneity. Studies on children with (developmental) disabilities were included if change in externalizing behavior was the primary outcome.

Relevant information was gathered from articles using a data extraction sheet (Table A in S3 File). Two reviewers checked the extracted data, and disagreements were resolved by consulting the articles and by discussion between the two authors (LW, TM). When information regarding sample size was not directly stated in the text, sample sizes were estimated from tables as exactly as possible. If only an overall sample size was stated, the sample size was divided into two to gain approximated values for the experimental and control groups. In exceptional cases, primary studies were consulted due to ambiguous information in meta-analyses. Additionally, if relevant information was lacking, further information was requested from authors of meta-analyses.

Some meta-analyses reported overall results as well as results for subsets of primary studies. In the case of meta-analyses on Triple P (delivering different models of parent interventions differing by intensity of intervention), only data for Levels 4 (broad focus parent training) and 5 (intensive family intervention) were included (if possible), because these levels target parents of children with more severe behavior problems and are more intense [26]. Some meta-analyses also reported effect sizes for statistically defined subsets of studies (e.g. only high-quality studies, without outliers, etc.). In such cases, overall effect sizes were included to obtain a larger data base. Furthermore, only non-weighted effect sizes were included, as synthesis of effects that are weighted for moderators or mediators can be very challenging [23].

### Risk of bias in included studies and quality ratings

The availability of risk of bias assessment and its quantification of influence on effect estimates in the included meta-analyses was registered.

Two reviewers independently rated the quality of every meta-analysis according to the PRISMA statement employing the PRISMA 2009 Checklist [27]. Each item was assessed on a 3-point Likert scale coding 0 (“item not fulfilled”), 1 (“item partially fulfilled”) or 2 (“item completely fulfilled”). Thus, quality ratings could range between 0 and 54. To ascertain inter-rater reliability, the intraclass correlation coefficient (ICC) was estimated for total scores using the SPSS version 22 [28]. The intraclass correlation was .886 (p < .001) and can thus be deemed excellent [29]. To depict a quality index, the means of the total scores of both raters were taken.

### Correction of primary study overlap

Given that overlap of primary studies might lead to distortion of results [24], overlap of primary studies was corrected for (see Appendix B in S1 File for formulae). The correction of primary study overlap had to be executed individually for each meta-meta-analysis.

To establish that each primary study contributed only once to the meta-meta-analysis, an adjusted value was estimated for each primary study that was included in multiple meta-analyses. For this purpose, the number of meta-analyses in which each primary study was included was determined. If primary studies could not be positively related to specific effect sizes in a meta-analysis, a conservative approach was adopted by correcting for all primary studies included in the meta-analysis. The inverse of this number was considered as the uniqueness of this study. For each meta-analysis, the uniqueness values of the included primary studies were summed up to determine the adjusted number of primary studies (k_adj_). Due to statistical reasons, meta-analyses with k_adj_ less than or equal to three were subsequently excluded and k_adj_ of the remaining studies were recomputed. Based on the adjusted number of primary studies (k_adj_), the standard errors for the effects from meta-analyses and finally the overlap-corrected weights for each meta-analysis were estimated.

### Meta-analytic procedure

We conducted random effects models using Comprehensive Meta-Analysis 2.0 (CMA) by Biostat [30] and R [31] package *metafor* [32].

For each meta-analysis, effect estimates were transformed to standardized mean differences (SMD). Only effect estimates that were based on at least two primary studies were used. If a meta-analysis stated multiple effect sizes for one outcome, these estimates were aggregated into a single effect size. Thus, each meta-analysis contributed only one effect size to the meta-meta-analysis. An overall SMD was computed from meta-analyses for each outcome. At this juncture, effect sizes of meta-analyses were weighted according to our colleagues [24] by the adjusted number of included primary studies k_adj_ to obtain an overall effect estimate accounting for primary study overlap (Appendix B in S1 File). In accordance with Cohen [33], 0.2 was interpreted as a small effect size, 0.5 as moderate, and 0.8 as large.

Heterogeneity was estimated via the Q test and I^2^ statistic. With regard to I^2^, heterogeneity can be interpreted as low (25%), moderate (50%) or high (75%) [34].

### Publication bias

Visual inspections of funnel plots displaying at least ten studies were carried out [15]. Furthermore, fail-safe *N*s (*N*_*fs*_) [35] were calculated for each outcome category to detect the number of studies with null results needed to reduce the observed result to a small effect size of 0.1.

## Results

### Search results

**Fig 1:**
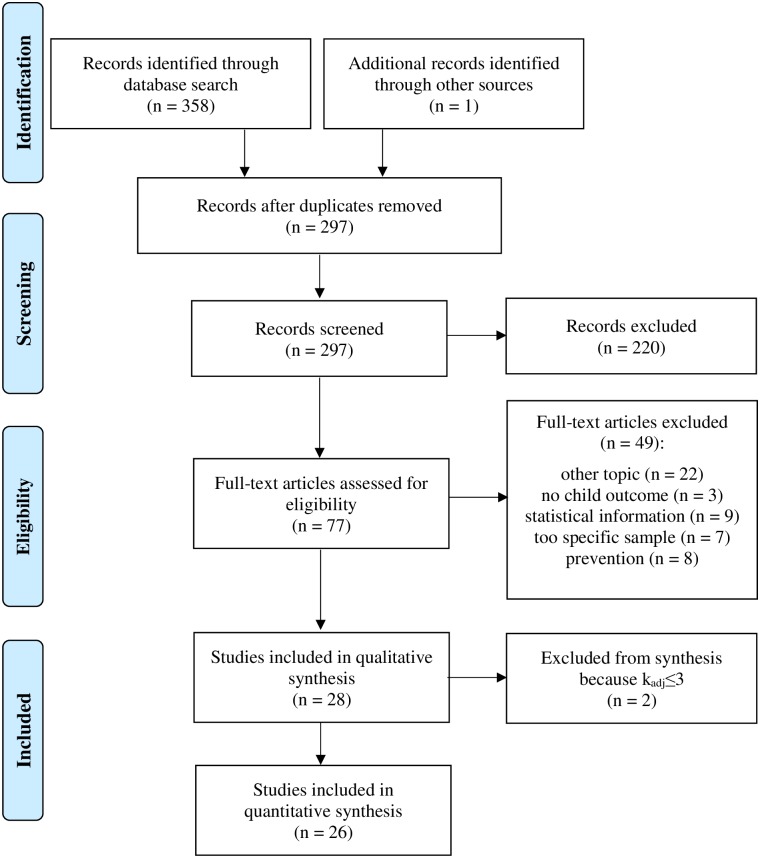
PRISMA flow diagram for studies included in and excluded from the meta-meta-analysis.

The qualitative synthesis encompasses the data of 28 meta-analyses (Fig 1). Due to the requirement of k_adj_ > 3, two additional meta-analyses [36,37] were ruled out, so that 26 meta-analyses were finally included in the quantitative synthesis.

### Study characteristics

The study characteristics of the included meta-analyses are illustrated in Table 1. On average, individual meta-analyses included 33.68 primary studies (SD = 34.84, range 4–156). While 20 meta-analyses exclusively considered controlled trials, 8 meta-analyses included controlled as well as uncontrolled trials. Therefore, effects were estimated on the one hand as comparisons of experimental and control groups and on the other hand on pre- to post-/follow-up-measures. While at post-measurement most meta-analyses solely included controlled trials (n = 23), data at follow-up measurement are based on controlled trials as well as pre- to follow-up-measures.

**Table 1:** Description of study characteristics.

Study	N primary studies^a^	Parent training	Total N	risk of bias assessment / influence	Clinical severity	Age of children	Comparison (FU-range)	Quality index
Buchanan-Pascall et al. [38]	16	Parent group programs (e.g. IY, Triple P)	1717	√ / yes	Majority of studies include children with diagnoses or sub-threshold symptoms	4–12 yrs.	EG-CG	45
Burkey et al. [39]	8	behavioral parenting interventions	1262	√ / no	3 treatment studies prevention interventions: 2 indicated, 2 selective, 1 universal	mainly ≤ 12 yrs. (1 study comprises youth > 12 yrs.)	EG-CG	49
Carr et al. [40]	10	Parents Plus programs	772 Pre-FU 374	√ / yes	clinical and community settings	1–16 yrs.	EG-CG Pre—FU (5–10 months)	31
Charach et al. [41]	13	PCIT, Triple P, IY, CBPT, HEAR, NFPP, other	558	√ / yes	clinically significant disruptive behavior	≤ 6 yrs.	EG-CG	38.5
de Graaf et al. [42]	15	Triple P (Level 4)	2574	NR	clinical (9 studies) and nonclinical (6 studies) range (ECBI)	2.18–7.7 yrs. (mean age in primary studies)	EG-CG Pre-FU (6–12 months)	34.5
Dretzke et al. [11]	24	structured & repeatable behavioral + nonbehavioral	1906	√ / NR	at least 50% conduct problems (above clinical cut-off point on validated measure and/or diagnosis of CD or ODD or informal diagnostic criteria)	≤ 12 yrs. mean age < 12 yrs. (49/57 studies)	EG-CG	40
Dretzke et al. [43]	37	structured & repeatable: behavioral approach (e.g. Triple P, Parents’ Plus) (31 studies) relationship approach (2 studies) elements of both behavioral and relationship approaches (4 studies)	2581	√ / NR	at least 50% conduct problems (above clinical cut-off point on validated measure and/or diagnosis of CD or ODD and/or description of behavioral problems or being disruptive)	mainly ≤ 12 yrs. (7 studies comprise youth > 12 yrs.)	EG-CG	39.5
Furlong et al. [8]	13	mainly IY, Barkley’s Parent Training Programme, GDVM, PMT, Workplace Triple P	1078	√ / yes	above clinical cut-off point on validated measure and/or diagnosis of CD or ODD	3–9 yrs. (M = 64 months)	EG-CG	49.5
Gardner et al. [44]	17	IY, TripleP, PCIT, PMTO	1558	√ / NR	conduct problems behavior scores above the clinical cut-off or referral to a specialist mental health center or diagnosis	3.5–8.4 yrs. (M = 5.6 yrs.)	EG-CG	43
Kok et al. [36] ^b^	4	behavioral (e.g. PCIT, Parents’ Plus, IY)	127	√ / NR	intellectual disabilities/ borderline intellectual functioning and psychiatric disorder	2–12 yrs.	EG-CG	41.5
Leijten, Melendez-Torres, et al [45]	Meta-analysis 1 156	parenting programs based on the principles of (social) learning theory (behavior management and/or relationship enhancement)	Meta-analysis 1 13478	√ / NR	treatment and prevention setting	Meta-analysis 1 2–9 yrs. M = 4.93	EG-CG FU EG-CG (1–36 months)	32.5
	Meta-analysis 2 41		Meta-analysis 2 5648			Meta-analysis 2 1–11 yrs. M = 5.54		
Lundahl et al. [12]	63	behavioral + nonbehavioral (e.g. PET, STEP)	3803	√ / no	mainly clinical, but also nonclinical symptoms	M = 81.42 months (SD = 42.23)	EG-CG FU EG-CG Pre-FU (1–72 months)	34
Maughan et al. [46]	79	behavioral	2083 + 1088 3171	√ / yes	target externalizing behavior (inclusion criteria)	3–16 yrs. (inclusion criteria)	EG-CG Pre-Post (FU EG-CG Pre-FU)	32.5
McCart et al. [47]	30	behavioral	1717	√ / yes (controlled for)	antisocial behavior mainly clinical setting (97%) diagnosis (6 studies/20%) at risk (24 studies/80%)	3–12 yrs. M = 65.28 months (SD = 24.96)	EG-CG	33.5
Menting et al. [48]	50	IY	4745	√ / no	Disruptive behavior 44% treatment studies 24% selective prevention 11% indicated prevention 10% not classified	3–9.2 yrs. (mean age in primary studies)	EG–CG	37
Mulqueen et al. [49]	8	behavioral (e.g. IY, PCIT, NFPP)	399	NR	clinical diagnosis of ADHD	3–5.36 yrs. (mean age in primary studies)	EG–CG	37.5
Nowak & Heinrichs [50]	55	Triple P	11797	√ / yes	31% of studies based on children with problems in clinical range	1–16 yrs.	EG-CG Pre-Post Pre-FU (3–36 months)	36.5
Piquero et al. [51]	78	parent training (e.g. IY, PCIT, Triple P) or Home visitation	13588	√ / yes	NR because of young age of children (<5 years) nonclinical range is probable	0–11 yrs.	EG-CG	31.5
Rimestad et al. [52]	14 (FU: 8)	mainly: IY, NFPP	1063	√ / no	mainly ADHD-diagnosis or ADHD-symptoms above clinical cut-off	3.47–5.23 yrs. (mean age in primary studies)	EG-CG (Post-FU 3–12 months)	49.5
Ruane & Carr [53]	11	Stepping Stones Triple P Level 4	694	√ / yes	all children with disabilities	1.60–9.79 yrs. (mean age in primary studies)	EG-CG	38.5
Sanders et al. [9]	101 (108 trials)	Triple P	16099 families	√ / yes	prevention and clinical range	0–18 yrs.	EG-CG Pre-Post FU EG-CG Pre-FU (2–36 months)	46
Serketich & Dumas [54]	26	BPT	M = 28.86 (SD = 18.36)	√ / yes	mainly clinical range	M = 6.05 yrs. (SD = 1.80)	EG-CG	24
Skotarczak & Lee [55]	11	behavioral (Parents’ Plus, Stepping Stones Triple P, IY)	540	√ / yes	Developmental disability Disruptive behaviors	4.11–8.54 yrs. (mean age in primary studies)	EG-CG	32.5
Tellegen & Sanders [56]	12	Stepping Stones Triple P	659 families	√ / no	all children with disabilities/ developmental disabilities; comorbid externalizing problems	1.5–17 yrs.	EG-CG Pre-Post	44
Thomas & Zimmer-Gembeck [13]	24	PCIT, Triple P	1519 (632 PCIT; 887 Triple P)	NR	clinical or borderline range	3.4–12 yrs.	EG-CG Pre-Post Pre-FU (4–12 months)	29.5
Van Aar et al. [57]	40	Parenting intervention (mainly: Triple P, IY, PMTO)	5782	√ / no	range of clinical severity (e.g. ten trials included only children with clinical levels of disruptive behavior; three trials included only children with non-clinical levels of problem behavior)	1.5–10.6 yrs. (mean age in primary studies)	EG-CG FU EG-CG (1 month– 3 yrs.)	48
Wilson et al. [16]	23	Triple P	1570	√ / NR	clinical and nonclinical range	2–13 yrs.	EG–CG	47
Zwi et al. [37]^b^	5	BPT, PFC	284	√ / NR	clinical diagnosis of ADHD/ hyperkinetic disorder	4–13 yrs.	EG-CG	43

BPT: Behavioral Parenting Training, CBPT: Community-Based Parent Training, CG: control group, EG: Experimental group; FU: Follow-up, GDVM: Webster-Strattons’ Group discussion videotape modelling training, HEAR: Helping Encourage Affect Regulation, IY: Incredible Years, N: sample size, NFPP: New Forest Parenting Program, NR: not reported, PCIT: Parent Child Interaction Therapy, PET: Parent Effectiveness Training, PFC: Parental Friendship Coding, PMT: Parenting Management Training, PMTO: Parent Management Training Oregon, STEP: Systematic Training for Effective Parenting.

^a^ number of primary studies included in meta-analyses. Due to primary study overlap, the sum of primary studies does not equal the specifications following.

^b^ not included in quantitative syntheses because k_adj_ <3.

23 studies contributed data for post measurement. Ten meta-analyses provided data on long-term effects, two of which could not be included in the meta-meta-analysis due to incomplete statistical information [46,52]. The eight included meta-analyses on average assessed follow-up between 2.88 (SD = 1.96) and 31.25 (SD = 20.51) months after completion of intervention (range 1–72 months). Overall, at post-measurement, 411 primary studies (reported in 436 articles) were included in the meta-meta-analyses and 135 at follow-up (reported in 148 articles).

The majority of the studies related to behavioral parent interventions, which are characterized by teaching parents effective behavioral strategies and skills to manage child behavior. Some studies [11,12,43] also included non-behavioral interventions. These interventions focus, for example, on parent-child communication or problem-solving strategies. Furthermore, some meta-analyses focused on specific parent-based interventions (Triple P, Stepping Stones Triple P, Incredible Years program, PCIT; e.g., [13,42,48,53,56]).

Basic descriptive statistics were often stated only partially. Although parental and child gender were not declared in all meta-analyses, one can act on the assumption that the majority of data was provided by mothers reporting on their sons (e.g., [40,50,56]). In most instances studies solely included parents of children under the age of 13 (range 0–18 years), only eight studies included youth older than 13 to some extent (e.g., [39,56]). The mean age could not be calculated due to missing information in the primary meta-analyses.

Child behavior overall as well as externalizing child behavior was assessed through parent reports (e.g. Child Behavior Checklist (CBCL)) or through observation of child behavior (e.g. Dyadic Parent–Child Interactive Coding System), and in several cases via teacher reports (e.g. Sutter-Eyberg Student Behavior Inventory (SESBI); Table B in S3 File).

### Risk of bias in included studies and quality ratings

The assessments of the potential risk of bias were conducted in 25 out of 28 meta-analyses (Table 1). Of those meta-analyses, 18 reported results concerning the influence of a potential risk of bias on study results (mainly publication bias), of which 12 found an influence.

Quality indices based on the quality ratings of every meta-analysis employing the PRISMA 2009 Checklist are illustrated in Table 1. The mean quality index was 38.88 (SD = 6.85, range 24–49.5). Overall, the quality of the included meta-analyses is satisfactory as the mean quality index exceeds the benchmark of two-thirds of the total score.

### Syntheses of results

Results are presented separately for child behavior overall and externalizing child behavior and are split into post-measurements and follow-up outcomes. Additional information is available in Tables C and D in S3 File.

### Child behavior overall

Data for post-measurements of child behavior based on parent report and observational data (overall effect) emerge from 23 meta-analyses. Meta-meta-analysis revealed a statistically significant and moderate effect for parent-based interventions (SMD 0.46, 95% CI 0.38 to 0.54, p = < .0001). Strong evidence of heterogeneity was observed (Q = 150.30, p = < .0001, I^2^ = 85.36%).

In a further step, separate effect sizes for parent report and observational data were examined. Meta-meta-analysis on parent report data revealed a statistically significant moderate effect size (SMD 0.51; 95% CI 0.39 to 0.64, p = < .0001), but with evidence of significant heterogeneity (Q = 124.66, p = < .0001, I^2^ = 84.76%).

The analysis of observational data revealed a significant moderate effect size (SMD 0.62, 95% CI 0.18 to 1.06, p = 0.0062) with no significant heterogeneity (Q = 2.72, p = 0.61, I^2^ = 0%).

Meta-meta-analysis of follow-up effects revealed a statistically significant moderate effect size for overall child behavior outcome (SMD 0.49, 95% CI 0.35 to 0.63, p = < .0001) with evidence of significant heterogeneity (Q = 65.10, p = < .0001, I^2^ = 89.25%).

Follow-up data on parent report of child behavior revealed a statistically significant moderate effect size (SMD 0.51, 95% CI 0.31 to 0.72, p = < .0001) with evidence of significant heterogeneity (Q = 40.22, p = < .0001, I^2^ = 87.57%).

Follow-up data on observational child behavior revealed a statistically significant moderate effect size (SMD 0.59, 95% CI 0.24 to 0.94, p = 0.0010) with no significant heterogeneity (Q = 0.02, p = 0.8922, I^2^ = 0%).

### Externalizing child behavior

Meta-meta-analysis on externalizing child behavior at post-measurement revealed a statistically significant moderate effect size (SMD 0.45, 95% CI 0.35 to 0.55, p = < .0001) with evidence of significant heterogeneity (Q = 71.26, p = < .0001, I^2^ = 84.56%).

Follow-up data on externalizing child behavior revealed a statistically significant moderate effect size (SMD 0.49, 95% CI 0.32 to 0.66, p = < .0001) with evidence of significant heterogeneity (Q = 26.76, p = < .0001, I^2^ = 88.79%.

### Risk of bias across studies

Due to the exclusion of unpublished studies, the risk of publication bias is certainly raised. On the other hand, including unpublished studies could also be a threat to validity, e.g. due to poor methodological quality [15].

The visual inspections of three funnel plots (S1, S2 and S3 Figs) display some asymmetry, thus suggesting a publication bias.

The fail-safe *N*s ranged between 10 and 96 (Figs 2–9) and indicate that at least quadruple to quintuple studies with null results are necessary to reduce effect sizes to a small level of 0.1. This relates to all conducted meta-meta-analyses and emphasizes the robustness of our findings.

**Fig 2:**
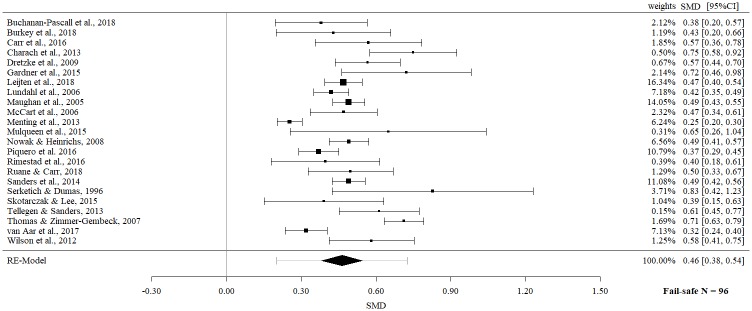
Meta-meta-analysis of child behavior overall (post-intervention).

**Fig 3:**
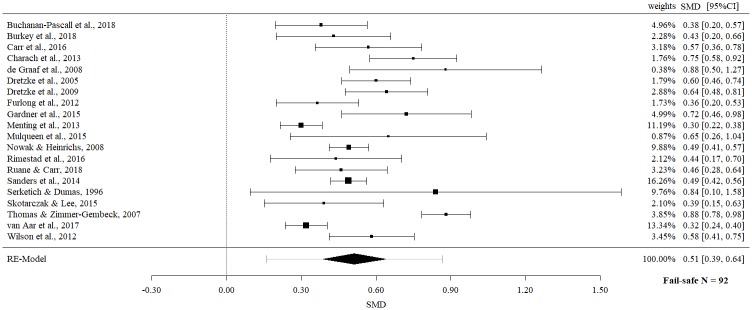
Meta-meta-analysis of parent report of child behavior overall (post-intervention).

**Fig 4:**
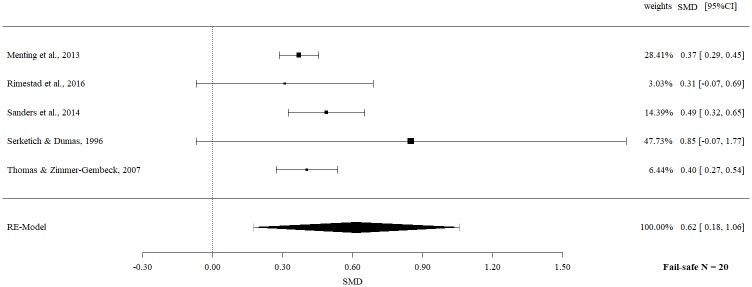
Meta-meta-analysis of observational data of child behavior overall (post-intervention).

**Fig 5:**
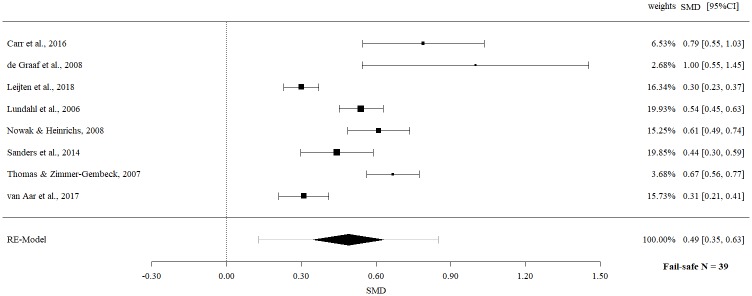
Meta-meta-analysis of child behavior overall (follow-up).

**Fig 6:**
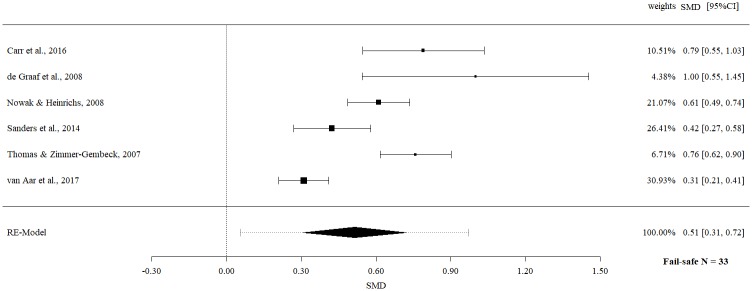
Meta-meta-analysis of parent report of child behavior overall (follow-up).

**Fig 7:**
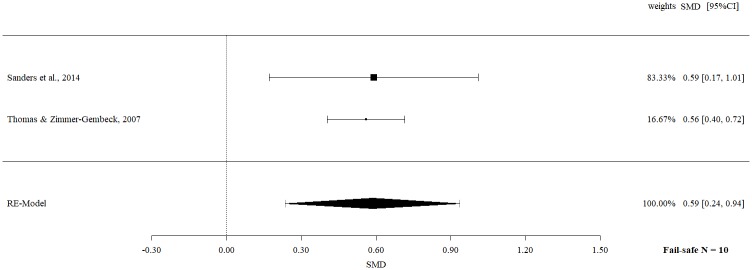
Meta-meta-analysis of observational data of child behavior overall (follow-up).

**Fig 8:**
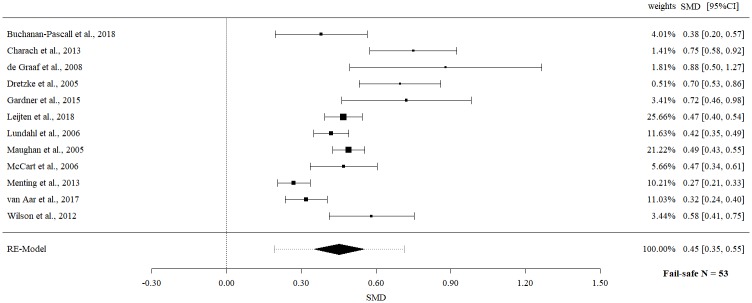
Meta-meta-analysis of externalizing child behavior (post-intervention).

**Fig 9:**
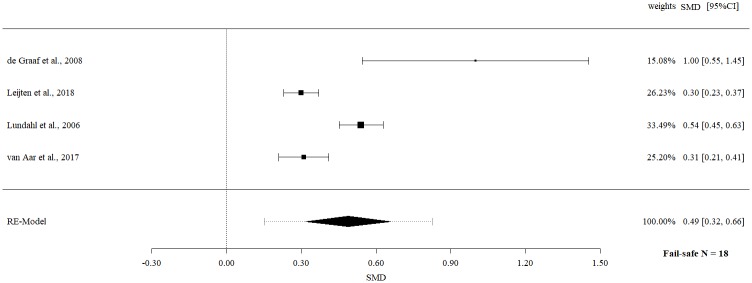
Meta-meta-analysis of externalizing child behavior (follow-up).

## Discussion

### Summary of evidence

To our knowledge, this is the first meta-meta-analysis on the effectiveness of parent-based interventions. Overall, the results suggest significant and moderate effects of parent-based interventions on child behavior that are stable over time. There is a great variability in the current literature regarding the effectiveness of parent-based interventions, ranging from small to large effects. Meta-analyses reporting large or small effects on child behavior are often based on a small number of studies [13,44], which reduces the probability of detecting the true effect [58,59]. The results shown in the current paper are based on 411 primary studies at post-measurement and on 135 studies at follow-up, and thus provide strong evidence for the true effect of parent-based interventions. The smallest effect (SMD = 0.25) was reported by Menting and colleagues [48] analyzing the effectiveness of the Incredible Years program in about 59 studies. Beyond that overall effect, severity of child behavior is reported to be a relevant moderator of effect size; subset analyses indicate a moderate effect size for the program in a treatment study context. These results are in line with the moderate overall effect detected in the present meta-meta-analysis. The meta-analyses by Serketich and Dumas [54] as well as de Graaf et al. [42] report large effect sizes. The meta-analysis by Serketich and Dumas [54] reaches the lowest score in the quality rating and therefore needs to be interpreted carefully. The meta-analysis by de Graaf et al. [42] analyzing the Triple P Program Level 4 shows good results in the quality rating and sets strict limits concerning outcome measure (only ECBI) and intervention (Triple P Level 4). Nevertheless, the effects of the included studies are very heterogenous (range 0.50–1.27) and subset analyses without outliers indicate a moderate effect size comparable to the results of the meta-meta-analysis.

Our results reach beyond the existing evidence by pointing to similar positive moderate and stable effects on child behavior for both parental and observational reports. Thus, they counteract common doubts concerning the evaluation of parent-based interventions. Often, the reliability of parental reports on child behavior in conjunction with parent-based interventions is called into question as due to a justification of efforts, child behavior changes might be reported more positively by parents [16,17]. The present meta-meta-analysis cumulates the existing results of five meta-analyses relating to 59 primary studies supporting parent training effectiveness via observational measures. These results refer to child behavior in general, future studies should also take observational measures of externalizing child behavior into account.

For the child outcome ‘child behavior overall’, different aspects of child behavior are assessed, including measures of disruptive behavior, conduct problems and ADHD symptoms [16,41] as well as measures of internalizing symptoms or prosocial behavior [9,48]. Although these results do represent the effect of parent-based interventions on child behavior overall, it remains unclear which aspects of child behavior are altered to which amount. To counteract this confusion, a separate effect for parent-based interventions based solely on externalizing symptoms was calculated. This more homogenous analysis revealed moderate effects on externalizing child behavior, representing the core symptoms of externalizing disorders in children. Additional analyses with respect to prosocial behavior or internalizing symptoms were not possible due to a lack of data.

### Future prospects

Future research should distinguish between different child outcome categories to allow for conclusions regarding the differential effectiveness of parent-based interventions. Since externalizing symptoms are the main target of parent-based interventions, a larger effect for this category can be expected compared to more distal child outcome categories such as internalizing symptoms or prosocial behavior. Combining these outcome categories leads to a somewhat biased appraisal of the effectiveness of parent-based interventions.

The impact of parent-based interventions on internalizing symptoms needs to be evaluated critically, since these often accompany childhood externalizing disorders [2]. Furthermore, research on the effectiveness of parent-based interventions for the treatment of children with internalizing disorders is scarce [10] and needs to be extended. Although some meta-analyses have already presented results on the effect of parent-based interventions on children’s prosocial behavior and reported small effects [48], further research is needed.

Another aim of future research should be to evaluate the comparative effectiveness of different interventions for children with externalizing behavior problems. In the present analyses, it was not possible to calculate separate effect sizes of parent-based interventions compared to different comparison groups (e.g. waitlist, no treatment, treatment as usual, active control group), as most studies aggregated these groups. Although effect sizes can be expected to decrease when comparing parent-based interventions to active control groups [16,43], studies with active control groups were included in our analysis in order to gain a more comprehensive impression of the effectiveness of parent-based interventions [41,44,48]. Confining the analyses to meta-analyses with only one type of comparison group would have restricted the database considerably, and would have excluded many relevant meta-analyses. Furthermore, some meta-analyses included studies with and without control groups (pre- to post-measures) for their effect estimation (e.g., [46,50,56]). Indeed, there is evidence that the quality of the included studies is more relevant than the inclusion of control groups [50].

Beyond that, moderator analyses considering possible variables influencing the effectiveness of parent-based interventions (e.g. symptom severity, child age, delivery format), or mediator analyses on changes in parenting behavior which mediate child behavior changes, are currently not possible and should be an aim for future research.

### Limitations

As combining various independent studies inevitably increases the risk of heterogeneity due to the heterogeneous design of the studies [15], the present results should be interpreted with caution [34]. In the present meta-meta-analysis, strict inclusion criteria were set to obtain homogeneity. However, since analyses are based on different meta-analyses, which for their part also include a wide array of primary studies, the achievable homogeneity is limited, as there is still considerable heterogeneity among primary studies (e.g. participants, interventions). A comparison with other meta-meta-analyses is restricted due to the limited number of second-order meta-analyses existing so far. Two previous meta-meta-analyses in the area of interventions for mental health problems did not find significant heterogeneity [24,60]. Nevertheless, meta-meta-analyses in the field of medicine or learning research did find heterogeneity [61,62].

Some results need further consideration. On the one hand the CI for observed behavior data at post-treatment and follow-up was quite wide, although no significant heterogeneity was found. This can on the one hand be due to different observational measures applied in different studies and on the other hand may be due to the small number of meta-analyses included in these analyses. Moreover, the limitation of small numbers of meta-analyses imbedded in analyses concerns all follow-up analyses. Therefore, the observational and follow-up results need to be interpreted with special caution.

Since the main focus of the current meta-meta-analysis was to evaluate the effectiveness of parent-based interventions as an intervention for treating children with externalizing behavior problems respectively externalizing disorders, we excluded studies that focused on the efficacy of parent-based interventions exclusively in preventive settings. However, parent-based interventions can include the parents of children as young as one to two years, and in this age range, the majority of interventions are preventive in nature. Thus, there were many meta-analyses which included prevention studies and clinical studies, and children with symptoms in the non-clinical and in the clinical range (e.g., [39,42,45,51,57]). As research indicates that effects of parent-based interventions in preventive settings are smaller, this could have negatively affected the magnitude of the derived effect sizes. Overall, the research suggests that the magnitude of initial problem severity affects the magnitude of the treatment effect, and for this reason, it is an important moderator with regard to the effectiveness of parent-based interventions [63]. Future studies should distinguish more carefully between prevention and clinical studies, or at least take special care to report more details of the included populations, especially concerning initial problem severity. In this way, the impact of initial problem severity on the effectiveness of parent-based interventions, and the possibility of a differential use of different forms of parent-based interventions could be examined.

Furthermore, it is to be taken into account that behavioral as well as non-behavioral parent-based interventions were included in the analyses. Research points to larger effects for behavioral parent-based interventions [64] thus the current analyses may underestimate the effects of behavioral parent-based interventions. Separate analyses for behavioral and non-behavioral parental interventions were not possible as some meta-analyses delivered combined results for both interventions. Nevertheless, most results rely on behavioral parent-based interventions, only three meta-analyses include behavioral as well as non-behavioral parent-based interventions. A related limitation concerns the combined analyses of interventions for children with ADHD, ODD and CD. On the one hand children with the various disorders could respond differently to parent-based interventions, on the other hand the diagnostic differentiation between these externalizing disorders especially in early childhood is difficult and considerable comorbidity exists. Moreover, most meta-analyses and primary studies include children with behavior problems or externalizing symptoms in general which precludes more differentiated analyses concerning ADHD, ODD and CD.

Another issue of detailed reporting in primary studies and meta-analyses relates to the description of child outcome categories. Unfortunately, meta-analyses often present somewhat heterogeneous child outcome measures, reporting, for example, the effect of parent-based interventions on child behavior, which represents a global measure of the achieved improvement, but also makes further and more detailed analyses impossible.

As meta-meta-analyses are still in their infancy, there are currently no guidelines or quality standards for such studies. When undertaking the present meta-meta-analyses, we drew on Cochrane recommendations and PRISMA guidelines formulated for meta-analyses and applied them as well as possible. Considering the risk of publication bias, one must take into account that the incomplete retrieval of unpublished studies certainly increases bias. On the other hand, the quality of results is weakened by the inclusion of unpublished studies [15]. Although we endeavored to extract data thoroughly, the lack of or insufficiently reported data in the primary meta-analyses limited our efforts, and extracted data might be imprecise in some cases. In case of doubt, data were derived conservatively (e.g. study overlap) to avoid overestimation of effects. To enable more precise analyses and correct interpretation of effects, we appeal to authors of meta-analyses to illustrate all relevant information precisely and to adhere to high quality standards (e. g. PRISMA guidelines [27]).

## Conclusions

Parent-based interventions are effective in treating children with externalizing behavior problems by means of reducing child problem behavior overall and externalizing child behavior in particular. Additionally, there are hints of cost-effectiveness [8] and positive long-term effects [51]. Based on these results, this meta-meta-analysis supports the classification of parent-based interventions as an evidence-based intervention for the treatment of children with externalizing behavior problems and disorders. Furthermore, there are some particular interventions that are already based on a broad empirical data base (e.g. Incredible Years, PCIT, Triple P). Goals for future research are to examine the differential effectiveness of parent-based interventions, and to close the still existing gap between research findings and clinical practice [65,66] by implementing this evidence-based intervention in practice.

Based on the solid data base indicating effectiveness of parent-based interventions, there should be a shift towards a broader offer of these interventions. Parents are crucial for the development of their children and are a relevant factor in the genesis of externalizing disorders. Therefore, the inclusion of caregivers in the treatment of child disorders is essential [66]. The long-term consequences of externalizing behavior problems and disorders are impairing for the affected children, their family and their environment, and are also expensive. It is thus crucial to enhance the mental health care for children with externalizing behavior problems and disorders, and we appeal to health care providers to make use of evidence-based parent-based interventions.

## Supporting information

S1 File Appendix A. Full electronic search strategy. Appendix B. Correction of primary study overlap according to Munder et al. (2013) [24].
(DOCX)

S2 File Protocol: PROSPERO, registration number CRD42016036486. (PDF)

S3 File Tables A-D. (DOCX)

S1 Table PRISMA checklist. (DOC)

S1 Fig Funnel plot displaying all studies on child behavior overall (post-intervention). (TIF)

S2 Fig Funnel plot displaying all studies on parent report of child behavior overall (post-intervention). (TIF)

S3 Fig Funnel plot displaying all studies on child externalizing behavior (post-intervention). (TIF)
