# Supplementary material for: Meta-meta-analysis on the effectiveness of parent-based interventions for the treatment of child externalizing behavior problems
Source: PLoS One. 2018 Sep 26;13(9):e0202855. doi: 10.1371/journal.pone.0202855 (PMC6157840; doi:10.1371/journal.pone.0202855)
Supplement: S1 File — Appendix A. Full electronic search strategy. Appendix B. Correction of primary study overlap according to Munder et al. (2013) [24]. (DOCX) [file pone.0202855.s001.docx]

**Appendix A.** **Full electronic search strategy.**

1. **PubMed (15 March 2016):**

All fields (No further limits were set.)

((meta-analysis) AND (((parent* intervention) OR parent* training) OR parent* program)) AND (((((children) OR preschool) OR toddler) OR childhood) OR infant)

1. **PsycInfo (15 March 2016):**

Abstracts only (No further limits were set.)

S1 AB parent* training OR AB parent* intervention OR AB parent* program

S2 AB children OR AB preschool OR AB toddler OR AB infant OR AB childhood

S3 AB meta-analysis

S4 (AB meta-analysis) AND (S1 AND S2 AND S3)

1. **MEDLINE (15 March 2016):**

All fields (No further limits were set.)

1 meta-analysis.af

2 (parent* training or parent* intervention or parent* program).af.

3 (children or preschool or toddler or childhood or infant).af.

4 1 and 2 and 3

**Appendix B. Correction of primary study overlap according to Munder et al. (2013) [24].**

1. **Formula for calculating uniqueness (U) of each primary study i:**

U_i_ = 1 / n.

n is the number of meta-analyses in which primary study i was included. The theoretical range of U_i_ is 1/n to 1.

1. **Formula for calculating adjusted number of primary studies (k_adj_) for each meta-analysis j:**

k_adj,j_ = ∑ U_i_ .

1. **Formula for calculating overlap-corrected weight (W) for each meta-analysis j:**

W_j_ = 1 / (τ² + SE_j_²).

τ² is the variation of the true effects and was estimated using the DerSimonian and Laird method ([Borenstein, Hedges, Higgins, & Rothstein, 2010](#_ENREF_1)).

1. **Formula for calculating the standard error for the effect from meta-analysis j (SE_j_):**

SE_j_ = 1 / (k_adj,j_ – 3)^1/2^.
