# Supplementary material for: Meta-meta-analysis on the effectiveness of parent-based interventions for the treatment of child externalizing behavior problems
Source: PLoS One. 2018 Sep 26;13(9):e0202855. doi: 10.1371/journal.pone.0202855 (PMC6157840; doi:10.1371/journal.pone.0202855)
Supplement: S2 File — (PDF) [file pone.0202855.s002.pdf]

Meta-meta-analysis of the effectiveness of parent training for the treatment of children with externalizing symptoms and disorders

Linda Weber, Tanja Mingebach, Inge Kamp-Becker, Katja Becker, Hanna Christiansen

Citation

Linda Weber, Tanja Mingebach, Inge Kamp-Becker, Katja Becker, Hanna Christiansen. Meta-meta-analysis of the effectiveness of parent training for the treatment of children with externalizing symptoms and disorders. PROSPERO 2016 CRD42016036486 Available from:
http://www.crd.york.ac.uk/PROSPERO/display_record.php?ID=CRD42016036486

Review question

How effective is parent training in treating externalizing symptoms and disorders in children.
How big is the impact of parent training on child behavior and on parental characteristics (parenting, parental mental health, parental perceptions and parental relationship).

Searches

We have searched the following electronic databases: MEDLINE, PubMed, and PsycINFO.
The search strategy included terms relating to typ of intervention, age of children and review type. In PsycINFO we only searched title and abstract. No further limits were applied.
The search terms will be re-run just before the final analyses and further studies retrieved for inclusion.

Types of study to be included

We have included English and German meta-analyses that focus on the effectiveness of parent based interventions for children with externalizing symptoms and disorders, not only in a preventive setting.

Condition or domain being studied

Childhood externalizing symptoms and disorders, child behaviour, parenting behaviour, parental mental health, parental perceptions and parental relationship.

Participants/population

Children with externalizing symptoms or disorders and their parents.

Intervention(s), exposure(s)

Parent based interventions for the treatment of mental disorders or behavior problems in children, but not in an exclusive preventive setting.
Examples include parent training delivered individually or in groups as well as interventions on parent-child-interaction.

Comparator(s)/control

Meta-analyses include active as well as inactive control groups and uncontrolled studies.

Primary outcome(s)

Changes in child behavior as well as in parenting behavior, parental mental health, parental perceptions and parental relationship as measured by parent report, independent report or observation (immediately after completion of parent training and at follow-up).

Secondary outcome(s)

Quality of included meta-analyses according to PRISMA Statement.

Data extraction (selection and coding)

Risk of bias (quality) assessment
Quality of every meta-analysis will be assessed independently by two reviewers according to PRISMA statement using the PRISMA 2009 Checklist (Liberati et al., 2009). Each item will be rated on a 3-point scale coding 0 (“item not fulfilled”), 1 (“item partially fulfilled”) or 2 (“item completely fulfilled”). Inter-rater reliability will be assessed.

Strategy for data synthesis
The effect estimates of each meta-analysis will be transformed to standardized mean effect (SMD) using a random-effects model. For each outcome an overall SMD will be computed from meta-analyses. Each meta-analysis contributes only one effect size to meta-meta-analysis. If a meta-analysis provides multiple effect sizes for one outcome, estimates will be averaged into a single effect size. Measures of consistency will be calculated.
To take overlap of primary studies into account, for each primary study the number of meta-analyses in which it is included is determined and an adjusted number of primary studies will be calculated for each meta-analysis. This adjusted number will serve as a weighting factor in the calculation of SMD.

Analysis of subgroups or subsets
If the necessary data are available, effect sizes will be estimated for different sources of information (parent report vs. observation). Because meta-analyses do not consistently report information on subgroups, further subgroup analyses are limited.

Contact details for further information
Mrs Weber
linda.weber@med.uni-marburg.de

Organisational affiliation of the review
Department of Child and Adolescent Psychiatry, Psychosomatics and Psychotherapy, Faculty of Human Medicine, Philipps-University Marburg

Review team members and their organisational affiliations
Mrs Linda Weber. Department of Child and Adolescent Psychiatry, Psychosomatics and Psychotherapy, Faculty of Human Medicine, Philipps-University Marburg
Ms Tanja Mingebach. Department of Child and Adolescent Psychiatry, Psychosomatics and Psychotherapy, Faculty of Human Medicine, Philipps-University Marburg
Professor Inge Kamp-Becker. Department of Child and Adolescent Psychiatry, Psychosomatics and Psychotherapy, Faculty of Human Medicine, Philipps-University Marburg
Professor Katja Becker. Department of Child and Adolescent Psychiatry, Psychosomatics and Psychotherapy, Faculty of Human Medicine, Philipps-University Marburg
Professor Hanna Christiansen. Philipps-University Marburg, Department of Clinical Child and Adolescent Psychology

Anticipated or actual start date
05 January 2015

Anticipated completion date
31 May 2016

Funding sources/sponsors
None

Conflicts of interest
None known

Language
English

Country
Germany

Stage of review
Review_Ongoing

Subject index terms status
Subject indexing assigned by CRD

Subject index terms
Child; Child Behavior Disorders; Humans; Parent-Child Relations; Parents

Date of registration in PROSPERO
15 March 2016

Date of publication of this version
15 March 2016

Details of any existing review of the same topic by the same authors

Stage of review at time of this submission

Stage	Started	Completed
Preliminary searches	Yes	Yes
Piloting of the study selection process	Yes	Yes
Formal screening of search results against eligibility criteria	Yes	Yes
Data extraction	Yes	No
Risk of bias (quality) assessment	No	No
Data analysis	No	No

Versions
15 March 2016

PROSPERO
This information has been provided by the named contact for this review. CRD has accepted this information in good faith and registered the review in PROSPERO. CRD bears no responsibility or liability for the content of this registration record, any associated files or external websites.
