# Supplementary material for: Meta-meta-analysis on the effectiveness of parent-based interventions for the treatment of child externalizing behavior problems
Source: PLoS One. 2018 Sep 26;13(9):e0202855. doi: 10.1371/journal.pone.0202855 (PMC6157840; doi:10.1371/journal.pone.0202855)
Supplement: S3 File — (DOCX) [file pone.0202855.s003.docx]

**Table A.** **Data extraction sheet.**

| **Study** | **parent training** | **child gender** | **child age** | **comparison** | **length of FU** | **outcome** | **rating** | **k** | **N (overall/ EG/ CG)** | **ES** | **CI / SE etc.** | **ES significance** | **statistical model** | **moderators** | **risk of bias assessment** |
| --- | --- | --- | --- | --- | --- | --- | --- | --- | --- | --- | --- | --- | --- | --- | --- |
|  |  |  |  |  |  |  |  |  |  |  |  |  |  |  |  |
|  |  |  |  |  |  |  |  |  |  |  |  |  |  |  |  |
|  |  |  |  |  |  |  |  |  |  |  |  |  |  |  |  |
|  |  |  |  |  |  |  |  |  |  |  |  |  |  |  |  |

CG: control group; CI: confidence interval; EG: experimental group; ES: effect size; FU: follow-up; k: number of primary studies; N: sample size; SE: standard error

Table B. Assessment instruments included in meta-analyses.

| **Study** | **Measures** |
| --- | --- |
| Buchanan-Pascall, Gray, Gordon, & Melvin, 2018 | CBCL-E, ECBI, SDQ-Conduct, DBD |
| Burkey et al. 2018 | CBCL, CAPES, ECBI, SDQ |
| Carr, Hartnett, Brosnan, & Sharry, 2017 | SDQ |
| Charach et al., 2013 | e.g. ECBI, CPRS, BASC, HSQ, PACS |
| de Graaf, Speetjens, Smit, de Wolff, & Tavecchio, 2008 | ECBI |
| Dretzke et al., 2009 | ECBI, CBCL, DPICS |
| Dretzke et al., 2005 | ECBI, CBCL, DPICS |
| Furlong et al., 2013 | CBCL, CBCL-DOF, C-II-Child, DISC-P, DPIS, DPICS, ECBI, HSQ,  LSDQ, MOOSES, PACS, PBQ, PDPQ, PDR, PPS-I CARE, PSI-child domain,  SCP, SDQ, SHP, SSQ, SSRS, TASB, Teacher PCSC, TRF |
| Gardner, Montgomery, & Knerr, 2015 | ECBI, CBCL-E, CBCL-ODD, SDQ-Conduct |
| Kok, van der Waa, Klip, & Staal, 2016 ^a^ | CBCL, DBC, SDQ |
| Leijten, Melendez-Torres, Gardner, van Aar, Schulz, & Overbeek, 2018 | NA (disruptive child behavior) |
| Lundahl, Risser, & Lovejoy, 2006 | e.g. ECBI, CBCL-E, PDR, DPICS, IBCS |

Table B (continued)

| **Study** | **Measures** |
| --- | --- |
| Maughan, Christiansen, Jenson, Olympia, & Clark, 2005 | NA (e.g. aggression, noncompliance, disruptive behavior) |
| McCart, Priester, Davies, & Azen, 2006 | NA (e.g. physical or verbal aggression, delinquency) |
| Menting, Orobio de Castro, & Matthys, 2013 | mainly ECBI (72%) NA (e.g. disruptive behavior, prosocial behavior) |
| Mulqueen, Bartley, & Bloch, 2015 | BASC, CBCL, CPRS, PACS, PKBS |
| Nowak & Heinrichs, 2008 | CAP, CBCL, CBQ-20, CDI, CMAS, CPC, DBC, ECBI, FOS / FOS-R-III, HCPC,  KINDL, PDR, PDRC, SBQ, SDQ, SEI, SESBI / SESBI-R |
| Piquero et al., 2016 | NA (conduct problems, delinquency, antisocial behavior) |
| Rimestad, Lambek, Christiansen, & Hougaard, 2016 | NYPRS, PKBS, PKBS-o/a, PACS, CBCL, ECBI, DBRS-odd, BPB, DSM-III  FOS-RII, PDT, PSA |
| Ruane & Carr, 2018 | CPC, CBCL, DBC, ECBI, SDQ, FOS |
| Sanders, Kirby, Tellegen, & Day, 2014 | CAP, CAPES, CBCL, CPC, DBC, ECBI, FBB, HCPC, KINDL,  LBC, PATFA, PDR, PDRC, PES, R-BPC, SBQ, SCAS, SDQ |
| Serketich & Dumas, 1996 | e.g. CBCL, ECBI |
| Skotarczak & Lee, 2015 | CBCL, ECBI, SDQ, DBC |

Table B (continued)

| **Study** | **Measures** |
| --- | --- |
| Tellegen & Sanders, 2013 | ECBI, SDQ, DBC, PDRC, CPC, FOS |
| Thomas & Zimmer-Gembeck, 2007 | ECBI, DPICS, CBCL, CTRS, SESBI, classroom observation,  DSM-ODD, BASC, PDR, FOS, HCPC, PSBC, SDQ |
| Van Aar, Leijten, Orobio de Castroc & Overbeek, 2017 | ECBI, CBCL-E, TRF-E, ITSEA-E, SDQ-Conduct, HCSBS-C, SSBS-C,  CABI-A, CABI-H, PRS-E, SCBE-E, KPC-E, R-BPC-C, SBQ-A, SBQ-E,  ECI-C, BCL-nc, PACS-A, PACS-C |
| Wilson et al., 2012 | ECBI, CBCL-E |
| Zwi, Jones, Thorgaard, York, & Dennis, 2011 ^a^ | ADDES-Home, ADDES-School, BASC, CBCL,  CPRS, CPRS-R-S-ADHD, DSAS, ECBI, SSRS, TRF |

ADDES-Home: Attention Deficit Disorder Evaluation Scale-Home Version, ADDES-School: Attention Deficit Disorder Evaluation Scale-School Version, BASC: Behavior Assessment System for Children, BCL-nc: Behavior check list-non-compliant, BPB: Behar Preschool Behaviour, CABI-A: Child adaptive behavior inventory-Aggression, CABI-H: Child adaptive behavior inventory-Hyperactivity, CAP: Child Attention Problems rating scale, CAPES: Child Adjustment and Parent Efficacy Scale, CBCL: Child Behavior Checklist, CBCL-ODD: Child Behavior Checklist – ODD subscale, CBCL-E: Child Behavior Checklist-Externalizing, CBCL-DOF: Child Behavior Checklist - Direct Observation Form, CBQ-20: Conflict Behavior Questionnaire, CDI: Children’s Depression Inventory, C-II-Child: Coder Impressions Inventory-Child, CMAS: Child Manifest Anxiety Scale (revised version), CPC: Care-giving Problem Checklist, CPRS: Conners’ Parent Rating Scale, CPRS-R-S-ADHD: Conners’ Parent Rating Scale-Revised Short Form-ADHD Index, CTRS: Conner’s Teacher Rating Scales, DBD: Disruptive behavior disorder rating scale, DBC: Developmental Behavior Checklist, DBRS-odd: Disruptive Behaviour Rating Scale Oppositionality Subscale, DISC-P: Diagnostic Interview Schedule for Children-Parent, DPIS: Dyadic Peer Interaction Scale, DPICS: Dyadic Parent-Child Interaction Coding System, DSAS: Dishion Social Acceptance Scale, DSM-III: parent ratings on Diagnostic and Statistical Manual of Mental Disorders, DSM-ODD: Diagnostic and Statistical Manual of Mental Disorders – Oppositional Defiant Disorder, ECBI: Eyberg Child Behavior Inventory, ECI-C: Early childhood inventory-Conduct, FBB: Fremdbeurteilungsbogen, FOS / FOS-RII / FOS-III-R: Family Observation Schedule / Revised- / -Revised III, HCPC: Home and Community Problem Checklist, HCSBS-C: Home and community social behavior scales-Conduct, HSQ: Home Situations Questionnaire, IBCS: Interpersonal Behavior Construct Scale, ITSEA-E: Infant-toddler social and emotional assessment–Externalizing, KINDL: Questionnaire for Measuring Health-Related Quality of Life in Children, LBC: Lifestyle Behaviour Checklist, KPC-E: Kohn’s problem checklist-Externalizing, LSDQ: Child Loneliness and Social Dissatisfaction Questionnaire, MOOSES: Multiple Option Observation System for Experimental Studies, NYPRS: New York Rating Scale Parent*,* PACS: Parental Account of Children’s Symptoms, PACS-A: Parental Account of Children’s Symptoms-Antisocial, PACS-C: Parental Account of Children’s Symptoms-Conduct, PATFA: Parent and Toddler Feeding Assessment, PBQ: Preschool Behavior Questionnaire, PDPQ: Parent Defined Problems Questionnaire, PDR: Parent Daily Report, PDRC: Parent Daily Report Checklist, PDT: Parent Directed Task, PES: Parenting Experience Survey, PKBS: Preschool and Kindergarten Behavior Scale, PKBS-o/a: Preschool and Kindergarten Behavior Scales Oppositional/Aggressive subscale, PPS-I CARE: Peer Problem-Solving-Interaction Communication-Affect Rating System, PRS-E: Parent rating scale-Externalizing, PSA: Parent Supervised Activit; PSBC: Problem Setting and Behavior Checklist, PSI-child domain: Parenting Stress Index - child domain, R-BPC: Revised Behaviour Problem Checklist, R-BPC-C: Revised Behaviour Problem Checklist-Conduct, SBQ: Social Behaviour Questionnaire, SBQ-A: Social Behaviour Questionnaire-Aggressive, SBQ-E: Social Behaviour Questionnaire-Externalizing, SCAS: Spence Children’s Anxiety Scale, SCBE-E: Social competence and behavior evaluation-Externalizing, SCP: Social Competence Scale - Parent Version, SDQ: Strengths and Difficulties Questionnaire, SDQ-Conduct: Strengths and Difficulties Questionnaire Conduct Scale, SEI: Coopersmith Self-Esteem Inventory child form, SESBI / SESBI-R: Sutter-Eyberg Student Bahvior Inventory(-Revised), SHP: Social Health Profile, SSBS-C: School social behavior scales-Conduct, SSQ: School Situations Questionnaire, SSRS: Social Skills Rating System, TASB: Teacher Assessment of Social Behavior, Teacher PCSC: Teacher Rating Scales of the Perceived Competence Scale for Young Children, TRF: Teacher’s Report Form, TRF-E: Teacher’s Report Form-Externalizing

^a^ not included in quantitative syntheses because k_adj_ <3

Table C. Outcomes for child behavior overall.

| Outcome | MA | k | SMD | lower  95% CI | upper  95% CI | p (SMD) | z | Q | df | p (Q) | I² (%) | Fail-Safe N (Orwin^a^) |
| --- | --- | --- | --- | --- | --- | --- | --- | --- | --- | --- | --- | --- |
| Post |  |  |  |  |  |  |  |  |  |  |  |  |
| Overall | 23 | 411 | 0.4637 | 0.3825 | 0.5448 | <.0001 | 11.1984 | 150.3015 | 22 | < .0001 | 85.36 | 96 |
| Parent report | 20 | 248 | 0.5136 | 0.3874 | 0.6397 | <.0001 | 7.9782 | 124.6649 | 19 | < .0001 | 84.76 | 92 |
| Observation | 5 | 59 | 0.6164 | 0.1753 | 1.0576 | 0.0062 | 2.7386 | 2.7197 | 4 | 0.6058 | 0 | 20 |
| FU |  |  |  |  |  |  |  |  |  |  |  |  |
| Overall | 8 | 135 | 0.4898 | 0.3489 | 0.6307 | <.0001 | 6.8131 | 65.1033 | 7 | < .0001 | 89.25 | 39 |
| Parent report | 6 | 107 | 0.5141 | 0.3077 | 0.7206 | <.0001 | 4.8819 | 40.2241 | 5 | < .0001 | 87.57 | 33 |
| Observation | 2 | 9 | 0.5861 | 0.2356 | 0.9366 | 0.0010 | 3.2777 | 0.0184 | 1 | 0.8922 | 0 | 10 |

MA: number of included meta-analyses; k: number of included primary studies – multiple articles reporting on the same sample are counted as one primary study; SMD: standardized mean difference; CI: confidence intervall; p: test for significance evaluated against .05; z: z-score; Q: test statistic for heterogeneity; df: degrees of freedom; I²: measure of degree of heterogeneity; FU: follow-up

^a^ number of studies with null results needed to bring observed result to a small effect size of 0.1

Table D. Outcomes for externalizing child behavior.

| Outcome | MA | k | SMD | lower  95% CI | upper  95% CI | p (SMD) | z | Q | df | p (Q) | I² (%) | Fail-Safe N (Orwin^a^) |
| --- | --- | --- | --- | --- | --- | --- | --- | --- | --- | --- | --- | --- |
| Post |  |  |  |  |  |  |  |  |  |  |  |  |
| Overall | 12 | 297 | 0.4529 | 0.3528 | 0.5531 | <.0001 | 8.8632 | 71.2591 | 11 | < .0001 | 84.56 | 53 |
| FU |  |  |  |  |  |  |  |  |  |  |  |  |
| Overall | 4 | 83 | 0.4887 | 0.3181 | 0.6593 | <.0001 | 5.6142 | 26.7598 | 3 | < .0001 | 88.79 | 18 |

MA: number of included meta-analyses; k: number of included primary studies – multiple articles reporting on the same sample are counted as one primary study; SMD: standardized mean difference; CI: confidence intervall; p: test for significance evaluated against .05; z: z-score; Q: test statistic for heterogeneity; df: degrees of freedom; I²: measure of degree of heterogeneity; FU: follow-up

^a^ number of studies with null results needed to bring observed result to a small effect size of 0.1
