# Supplementary figures and images for: Meta-meta-analysis on the effectiveness of parent-based interventions for the treatment of child externalizing behavior problems
Source: PLoS One. 2018 Sep 26;13(9):e0202855. doi: 10.1371/journal.pone.0202855 (PMC6157840; doi:10.1371/journal.pone.0202855)

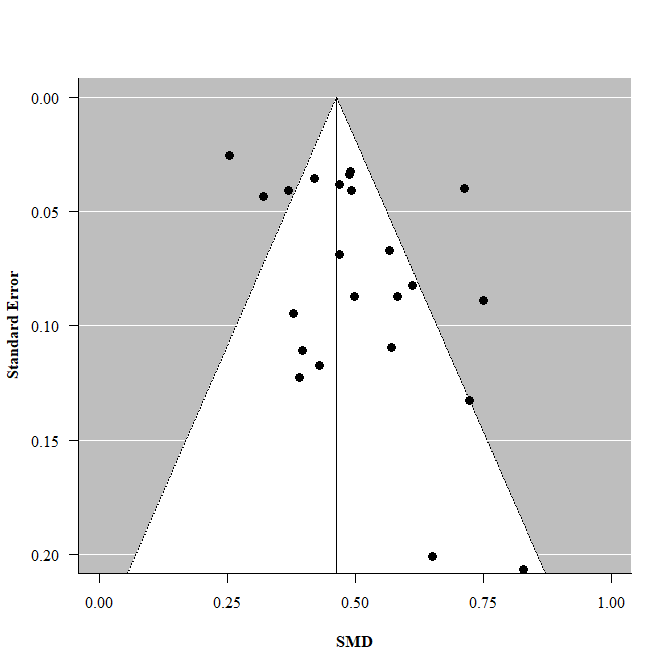

Supplement: S1 Fig — (TIF) [file pone.0202855.s005.tif]

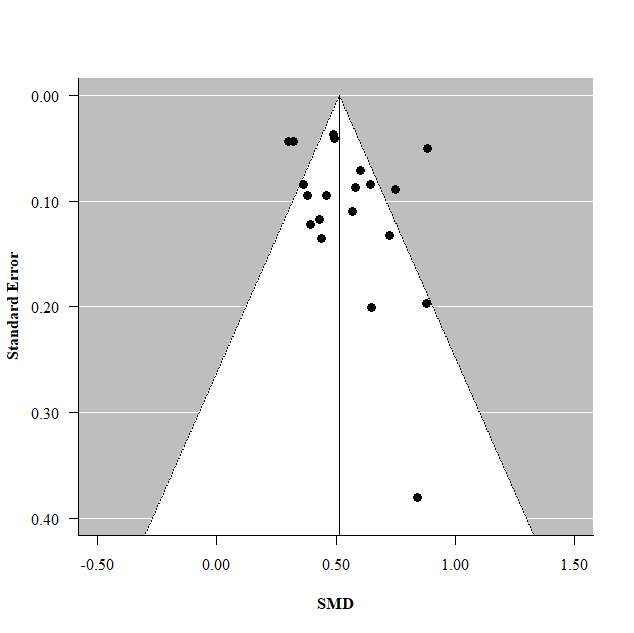

Supplement: S2 Fig — (TIF) [file pone.0202855.s006.tif]

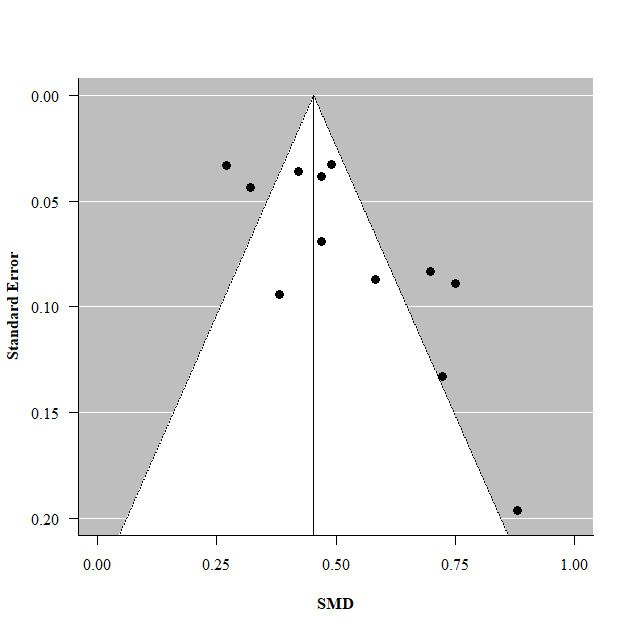

Supplement: S3 Fig — (TIF) [file pone.0202855.s007.tif]
